# Notes and News

**Published:** 1905-03-18

**Authors:** 


### Notes and News.

The Duchess of Albany will attend the annual meeting of the governors and members of the National Hospital for the Relief and Cure of the Paralysed and Epileptic at the hospital, Queen Square, Bloomsbury, on Tuesday, March 21st, at 3 p.m.

The Royal Commission on the Care and Control of the Feeble Minded recommenced its sittings on March 5th, under the chairmanship of Lord Radnor.

Sir Samuel Scott, Bart., M.P., has been elected chairman of the Committee of Management of Queen Charlotte's Hospital in succession to the late Earl of Hardwicke.

A dinner will be given in honour of Dr. F. Roberts by his former house physicians. Dr. Roberts has held the post of physician at University Cottage Hospital for a quarter of a century.

The arrangements to conduct members of the International Congress of Medicine to Lisbon by sea, next year, are sure to give satisfaction, as accommodation on board will be available during the duration of the Congress.

The annual Board of Governors of St. John's Hospital for Diseases of the Skin will be held in the Pinafore Room of the Savoy Hotel on Tuesday, March 28th, at 4.30. The Earl of Chesterfield will preside.

A correspondent sends the following amusing advice on prescribing, which is quoted from the work of a well-known member of the medical profession: "A patient at a metropolitan hospital goes away best satisfied when he is given something to drink out of a bottle. The drinking, according to ancient ritual, must not be less often than three times a day and the ceremony must have some reference to meals. The draught to be efficient should be coloured. It must have a marked odour so that he may invite his friends to smell it. It should be loathsome to the taste, so that the taking of it may call for some heroism. Above all it needs to possess an evil looking sediment, which will require a formal shaking of the phial."

The late Mr. E. W. Davidson, of Mossley Hill, has bequeathed the majority of his estate of £112,233 to charities, amongst which are the Royal Infirmary and the Royal Southern Hospital, Liverpool.

A conference was held last week at Buckingham Palace, between the representatives of the War Office and the Red Cross Society, the National Aid Society, and St. John's Ambulance Association, to consider the question of improving the medical corps systems in the field, through the closer co-operation amongst the voluntary societies and these with the War Office. Another and larger meeting is proposed later on.

In the House of Lords last week the Duke of Northumberland asked the question whether it was the intention of the Government to provide for the revaccination of children at school age. Lord Kenyon replied that inquiry had shown that compulsory revaccination was not practicable in this country, and therefore the reply was in the negative. He spoke of the success of the Act of 1898 in reducing small-pox.

The Liverpool School of Tropical Medicine have created a lectureship in Economic Entomology and Parasitology, and elected Mr. R. Newstead to be the first occupant of the chair. Mr. Newstead has had much practical experience of insects injurious to man, animals, and plants, under the Board of Agriculture and in the Colonies. He acted as assistant to the late Miss Ormerod, and was External Examiner in Entomology at the Edinburgh University.

We recently referred to a meeting called to establish a home of recovery in the country for patients who require a continuance of medical treatment and nursing on leaving the hospitals. To show how much such institutions are needed, and that the Convalescent Home does not meet the case, we quote from a paragraph appearing in the report of one of these institutions:—"Several unsuitable cases have been recommended for admission during the year . . . the homes are not intended as a place for the cure of disease. . . . It will be to the advantage and in the interests of those patients suffering from the diseases named in the certificate and in the recommendation lines that they should not be sent to the Homes in a weak state of health."

At a meeting of the Council of Royal College of Surgeons of England last week, a report was received from the board of examiners in dental surgery recommending, for adoption by the council, synopses indicating the range of examination in the subjects of anatomy, physiology, surgery, and surgical pathology for candidates for the licence in dental surgery. This report was adopted. Mr. H. T. Butlin, vice-president, was appointed Bradshaw lecturer for the ensuing year, and the president reported that Sir Gilbert Blane's medals had been awarded to Surgeon S. T. Reid, his Majesty's ship *Vestal*, 1901-2, and Surgeon R. W. G. Stewart, M.B., his Majesty's ships *Latona* and *Thames*, 1903. It was determined to hold a Fellows' subscription dinner at the college on the day of the council election in July next.

